# Open access in 2011: a publisher's perspective

**DOI:** 10.1186/1742-4690-8-S2-O25

**Published:** 2011-10-03

**Authors:** Nandita A Quaderi

**Affiliations:** 1 BioMed Central, 236 Gray's Inn Road, London, UK

## 

Over the last decade the open access (OA) publishing model has secured itself as an integral part of the biomedical publishing landscape. Furthermore, as a growing number of funding agencies introduce OA mandates, it is becoming increasingly important for researchers and institutions to comply with these policies. This presentation will summarise the different routes to achieving open access and give an overview of recent progress and developments in OA publishing.

One of the key benefits of OA is the removal of subscription barriers to published research - this is especially true for communities that suffer from limited visibility for their research output and/or access to the necessary scholarly journals. BioMed Central is taking a lead role in raising awareness of the advantages of OA, and making OA an affordable publishing option, for researchers based in Africa and other parts of the developing world. The second part of this presentation will focus on these initiatives.

